# Hydrostatic Pressure Modulates Intervertebral Disc Cell Survival and Extracellular Matrix Homeostasis via Regulating Hippo-YAP/TAZ Pathway

**DOI:** 10.1155/2021/5626487

**Published:** 2021-06-16

**Authors:** Yiyang Wang, Baoshuai Bai, Yanzhu Hu, Haoming Wang, Ningyuan Liu, Yibo Li, Pei Li, Guangdong Zhou, Qiang Zhou

**Affiliations:** ^1^Department of Orthopedics, The Third Affiliated Hospital of Chongqing Medical University, Chongqing 401120, China; ^2^Tissue Repairing and Biotechnology Research Center, The Third Affiliated Hospital of Chongqing Medical University, Chongqing 401120, China; ^3^National Tissue Engineering Center of China, Shanghai 200241, China; ^4^Department of Plastic and Reconstructive Surgery, Shanghai Ninth People's Hospital, Shanghai Jiaotong University School of Medicine, Shanghai Key Laboratory of Tissue Engineering, Shanghai 200011, China; ^5^Research Institute of Plastic Surgery, Wei Fang Medical College, Wei Fang 261053, China; ^6^Department of Orthopedics, Three Gorges Central Hospital of Chongqing University, Chongqing 404000, China; ^7^Department of Orthopedics, General Hospital of Central Theater Command, Wuhan 430000, China

## Abstract

Established studies proved that hydrostatic pressure had multiple effects on the biological behavior of the intervertebral disc (IVD). However, the conclusions of the previous studies were inconsistent, due to the difference in hydrostatic loading devices and observing methods used in these studies. The current study is aimed at investigating the role of dynamic hydrostatic pressure in regulating biological behavior of the notochordal nucleus pulposus (NP) and fibrocartilaginous inner annulus fibrosus (AF) and its possible mechanism using our novel self-developed hydrostatic pressure bioreactor. The differences in the biological behavior of the rabbit IVD tissues under different degree of hydrostatic pressure were evaluated via histological analysis. Results revealed that low-loading dynamic hydrostatic pressure was beneficial for cell survival and extracellular matrix (ECM) homeostasis in notochordal NP and fibrocartilaginous inner AF via upregulating N-cadherin (N-CDH) and integrin *β*1. In comparison, high-magnitude dynamic hydrostatic pressure aggravated the breakdown of ECM homeostasis in NP and inner AF via enhancing the Hippo-YAP/TAZ pathway-mediated cell apoptosis. Moreover, inner AF exhibited greater tolerance to physiological medium-loading degree of hydrostatic pressure than notochordal NP. The potential mechanism was related to the differential expression of mechanosensing factors in notochordal NP and fibrocartilaginous inner AF, which affects the fate of the cells under hydrostatic pressure. Our findings may provide a better understanding of the regulatory role of hydrostatic pressure on the cellular fate commitment and matrix metabolism of the IVD and more substantial evidence for using hydrostatic pressure bioreactor in exploring the IVD degeneration mechanism as well as regeneration strategies.

## 1. Introduction

The intervertebral disc (IVD) consists of two compartments, nucleus pulposus (NP) and annulus fibrosus (AF) [1], which connects the adjacent bony vertebral bodies. NP tissue is a type of gelatinous structure, containing collagen fibrils and proteoglycan molecules, primarily aggrecan [2]. NP is surrounded by AF, composed of type I and II collagen fibrils, arranged at alternating oblique angles to form concentric lamellae [3]. IVD tissue is formed by NP and AF, but they are derived from different embryonic structures. NP is derived from the notochord, while AF is derived from the somites [1, 4, 5]. In addition, proteoglycan molecules are more abundant in NP and inner AF than outer AF [3]. The high abundance of hydrated proteoglycan helps buffer the compressive loading of the spine and keep the collagen ultrastructure together within the tissues [6]. Thus, the cells in the NP and inner AF live in a unique microenvironment with hydrostatic pressure.

Some studies that reported the effects of hydrostatic pressure on IVD tissues or cells obtained from different species. However, the conclusions of the studies are not entirely consistent, due to the different quality of hydrostatic loading devices and observing methods used in the studies. It has been reported that the “physiological intensity (0.35-0.75 MPa)” of hydrostatic pressure acts as an anabolic factor for NP and AF cells, owing to the stimulation of proteoglycan synthesis [7–9]. In contrast, excessive pressure aggravates the catabolic metabolism of proteoglycan in NP and AF cells [7–9]. Another study reported that the proteoglycan synthesis is inhibited at 0.35 MPa compared to atmospheric pressure for both NP and AF cells harvested from the lumbar IVD tissue of dogs [10]. However, collagen synthesis is stimulated in NP cells but inhibited in AF cells [10]. The discrepancy of these experimental results may be caused by the reason that the cells used in the prior studies are cultured in dishes or alginate. The matrix stiffness, elastic modulus, and penetration of nutrients are quite different from the natural matrix of IVD [7, 8, 10]. Besides, NP tissue goes through a transition from a notochordal to a fibrocartilaginous one, which accompanies changes in cell type from notochordal to fibrochondrocyte-like cell. In humans, the transition usually is completed before twenty years old [11]. Whereas in some animals, such as rabbits or dogs, the notochordal NP is permanently preserved in some proportion [5, 12–14]. According to some studies, the response of mature nucleus pulposus and notochordal cells to the hydrostatic pressure is quite different [15, 16]. In addition, it has been reported that hydrostatic pressure could induce the transition of notochordal NP to fibrocartilaginous NP [15–17].

Given the different cell phenotypes in IVD, the current study was conducted to demonstrate the response of notochordal NP and fibrocartilaginous inner AF to graded hydrostatic pressure. To that end, we set up a novel hydrostatic pressure tissue culturing model to investigate the different biological behavior of the two cohesive tissues in the same IVD sample. We cut off the cartilage endplates and exposed the NP and inner AF tissue of the rabbit lumbar IVD, which is a frequently used model for studying the IVD biological behavior and degeneration progress [12]. Next, the treated IVD were cultured in our newly developed hydrostatic pressure bioreactor for four weeks. Our previous study has demonstrated that the physiological degree of hydrostatic pressure could stimulate cell proliferation and ECM production of articular cartilage, which confirmed the feasibility and reliability of our hydrostatic pressure culturing system [18]. A previous study indicated that N-CDH and integrin-mediated adhesion interaction modulated YAP-TAZ associated mechanosensing and fate commitment of cells [19]. Our previous study elucidated that N-cadherin (N-CDH) played as a protective role in NP cells against overloaded compression [20]. Additionally, some studies reported that fibronectin binding to integrin *β*1 led to elevate the expression of apoptosis-related proteins [21, 22]. N-CDH and integrin-associated regulatory roles may involve histologic and biochemical alterations of disc cells induced by compression-loading stress. Hence, in the current study, we aim to figure out the differences and potential mechanism in the biological behavior of notochordal NP and fibrocartilaginous inner AF under different degree of hydrostatic pressure via investigating the histological feature, cellular survival, and ECM metabolism. The present study may provide a novel hydrostatic pressure-based IVD regeneration strategy and potential regulating target to treat compressive loading-related IVD degeneration.

## 2. Materials and Methods

### 2.1. Source of Animals

Mature male New Zealand white rabbits (160-week-old, weighing 3.5-3.8 kg) were used in the current study. The lumbar spines were obtained aseptically from the rabbits after euthanasia by an excess dose of sodium pentobarbiturates.

### 2.2. Tissue Dissection and Culture

The spine motion segments were sharply cut at the proximal and distal vertebrae in the axial parallel plane close to the adjacent endplates and washed with phosphate-buffered saline (PBS) five times in the tissue culture dishes (Jet Biofil, China). Then, the cartilage endplates of the IVDs were sharply cut to expose the NP and inner AF tissues. After that, the treated IVDs were moved to the culture chamber of our self-developed dynamic hydrostatic loading organ culture bioreactor. The tissues were cultured in the chamber with complete culture medium (DMEM/F-12 (Gibco, USA) containing 10% fetal bovine serum (FBS, Gibco, USA) and 1% penicillin/streptomycin (Gibco, USA)) and incubated (5% carbon dioxide, 37°C) for four days before exerting hydrostatic pressure. Culturing media were replaced every four days.

### 2.3. Hydrostatic Pressure Exerting Protocol

During the pressure performing regime, culture chambers were transferred to our custom-made dynamic hydrostatic pressure bioreactor. We set the pressure apparatus to provide intermittent hydrostatic pressure. Briefly, every dynamic hydrostatic pressure exerting cycle contained a 30 seconds pressure exerting phase and a 30 seconds pressure releasing phase. The samples were assigned into the following groups: the control group (hydrostatic pressure = 0 MPa) and the gradient dynamic hydrostatic pressure exerting groups (hydrostatic pressure = 0.5 MPa, 0.8 MPa, 1.0 MPa, 3.0 MPa, 5.0 MPa). Each group was exerted dynamic hydrostatic pressure for 2 hours per day and then transferred to the incubator (5% carbon dioxide, 37°C). The culture medium was replaced every four days. IVD specimens were collected for further experimental testing after 30 days of culture.

### 2.4. Histological Haematoxylin and Eosin (HE) Staining

The treated samples were harvested and fixed with 4% paraformaldehyde, embedded in paraffin, and then cut into 5 *μ*m per section. Then, the tissue sections were stained with haematoxylin and eosin. The stained sections were observed and scanned under an optical microscope (Olympus, Japan).

### 2.5. Alcian Blue Staining

Alcian blue staining was used to detect the ECM glycosaminoglycan deposition. Briefly, each tissue section was incubated in the 0.2% alcian blue solution before rinsing with deionized water. Then, the stained cells were mounted and observed under an optical microscope (Olympus, Japan).

### 2.6. Sulfated Glycosaminoglycan (sGAG) Quantification

For quantitative analysis of ECM, the sGAG content of each group was quantified by the dimethyl methylene blue chloride (Sigma-Aldrich, USA). Total sGAG was precipitated by the 0.98 mol/L guanidinium chloride solution. After that, the optical density (OD) was detected at 595 nm. The sGAG contents were determined according to the OD value and the standard curve.

### 2.7. Fluorescent TdT-UTP Nick End Labeling (TUNEL) Assays

TUNEL assays were performed to detect apoptosis of target cells with the One Step TUNEL Apoptosis Assay Kit (Beyotime, China) according to the manufacturer's instructions. Tissue sections were firstly treated with Triton X-100 (0.3%) for 5 minutes at room temperature. Then, the cells were treated with TUNEL for 1 hour at 37°C at the dark. The sections were imaged via fluorescence microscopy (Leica, Germany) after the DAPI staining.

### 2.8. Immunohistochemistry (IHC)

To further assess the synthesis of the ECM, IHC staining was utilized to detect the aggrecan, collagen type I and II content. After rehydration, tissue sections were blocked by goat serum, treated with hyaluronidase (0.8%) for 20 minutes at 37°C, and then incubated with aggrecan, type I and II collagen antibody (1 : 100, Abcam, UK) for 60 minutes. After washing in PBS, biotinylated secondary antibody (1 : 100, Dako, Denmark) was applied for 30 minutes, washed in PSB, and treated with avidin-biotin complex reagents. Colour was developed using 3,3-diaminobenzidine reagents (Dako, Denmark), and the sections were counterstained with Harris's haematoxylin. The average optical density (AOD) of five randomly selected visual fields (per immunohistochemical slice) under high magnification (400x) was measured using the Image-J analysis system.

### 2.9. Immunofluorescence Staining

After rehydration, tissue sections were blocked by goat serum, treated with hyaluronidase (0.8%) for 20 minutes at 37°C, and then incubated with N-CDH antibody, integrin*β*1antibody (1 : 100, Abcam, UK), and YAP-TAZ antibody (1 : 100, Santa Cruz, USA) overnight at 4°C. Next, after an additional wash step, the sections were incubated with the fluorescent secondary antibody (1 : 1000; Proteintech, China) for 2 hours at room temperature, protected from light. The sections were then stained with DAPI and imaged using fluorescence microscopy (Leica, Germany).

### 2.10. Tissue Protein Extraction and Western Blotting

After being ground in liquid nitrogen, the tissues were lysed with RIPA lysis buffer containing 1% PMSF (Beyotime, China) for 30 minutes at 4°C. Then, the lysates were centrifuged at 12000 × g for 8 minutes at 4°C. The protein samples were subjected to SDS-polyacrylamide gel electrophoresis and transferred by electroblotting to PVDF membranes. The bands were then incubated with the primary antibodies (anti-N-CDH, anti-integrin-*β*1 (1 : 1000; Abcam, UK), anti-YAP (1 : 1000; Santa Cruz, USA), anti-Caspase3, and anti-GAPDH (1 : 500; Proteintech, China)) overnight at 4°C. After the bands were washed with TBST thrice, they were incubated with the secondary antibody for 80 minutes at room temperature. Being washed with TBST thrice, the intensity of the blots was detected by the Image Lab software (Bio-Rad, USA).

### 2.11. Statistical Analysis

All data were analyzed using GraphPad Prism (version 6.0, GraphPad Software, USA), and presented as mean ± standard deviation with *n* = 3. Two-tailed Student's *t*-test was used to assess the statistical significance of results (*P* < 0.05).

## 3. Results

### 3.1. Effect of Hydrostatic Pressure on Histomorphology and Glycosaminoglycan Synthesis of the Rabbit Notochordal NP and Fibrocartilaginous Inner AF

**Figure 1:**
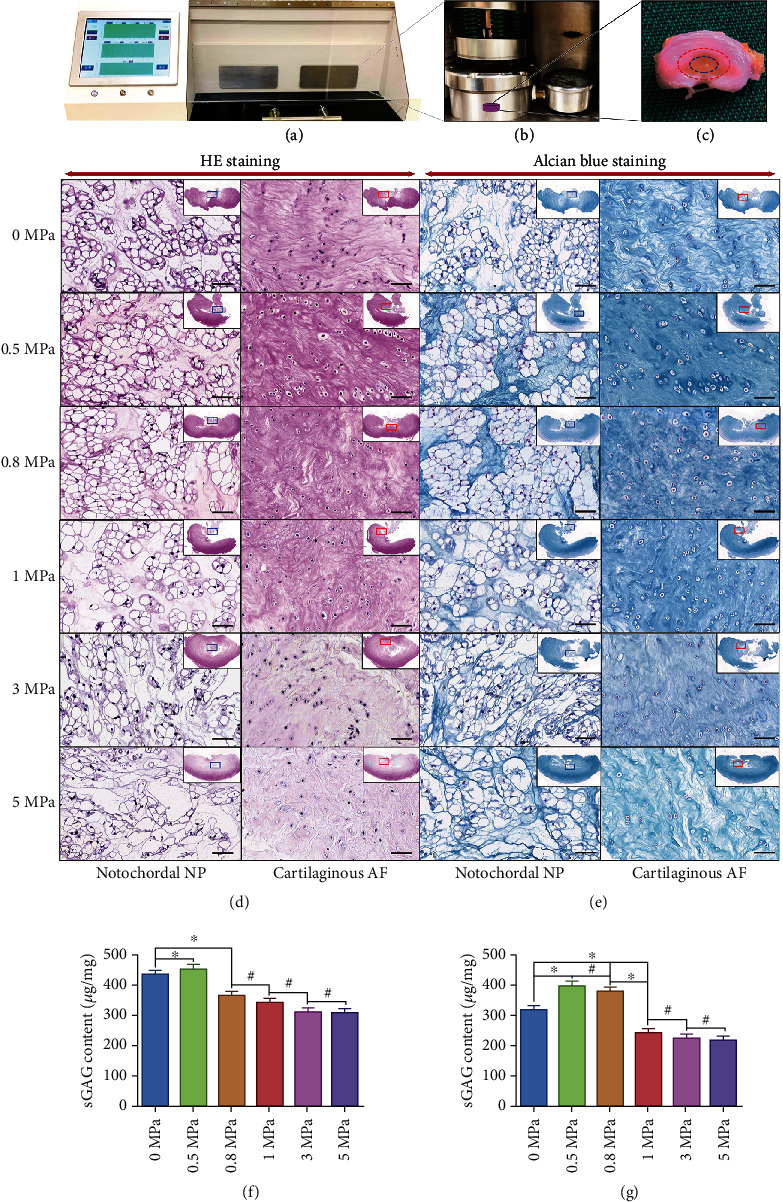
Effect of hydrostatic pressure on histomorphology and glycosaminoglycan synthesis of the rabbit notochordal NP and fibrocartilaginous inner AF: (a) appearance of the self-developed hydrostatic pressure bioreactor and touch-screen control system; (b) appearance of the tissue culture chamber of hydrostatic pressure bioreactor; (c) gross view of the endplate-removed rabbit IVD: the area in the blue circle indicates the notochordal NP, and the area between the red and blue circles indicates the fibrocartilaginous inner AF; (d) HE staining of the notochordal NP and fibrocartilaginous inner AF under graded hydrostatic pressure (200x); (e) alcian blue staining of the notochordal NP and fibrocartilaginous inner AF under graded hydrostatic pressure (200x); (f) quantification of the notochordal NP sGAG under graded hydrostatic pressure; (g) quantification of the fibrocartilaginous inner AF sGAG under graded hydrostatic pressure. ^∗^P < 0.05. ^**#**^P > 0.05. Scale bar = 100 *μ*m.

According to the study's design, we need to use the IVD simultaneously contains notochordal NP and fibrocartilaginous inner AF as our researching subject. We harvested IVD from twelve mature rabbits of same age and sex, and five of the rabbits maintained entire notochordal NP with many vacuoles of various sizes. The occurrence rate of the notochordal NP in mature rabbits and its tissue gross and histological morphology was consistent with the previous studies [12, 23]. Next, the IVD tissues cohesively with notochordal NP and fibrocartilaginous inner AF were divided into six groups and cultured in chambers with different levels of hydrostatic pressure exerted by our self-developed bioreactor (Figures 1(a) and 1(b)). After 30 days in vitro culture, the histological morphology of the NP and inner AF tissue revealed evident diversity. As is shown in Figure 1(d), the notochordal NP appeared as a bubbled island consisted of a cell-vacuole complex in the homogeneous basophilic ECM. The inner AF exhibited a fibrocartilaginous morphology, with cells smaller in size than those of the notochordal NP tissue but larger in size and more rounded than those of the outer AF (Figure 1(d)). Moreover, HE staining results indicated that the morphology of the notochordal NP and inner AF cells was relatively normal in groups of tissues under ≤1.0 MPa hydrostatic pressure (Figure 1(d)). However, when the grade of hydrostatic pressure was raised to ≥3 MPa, the cytoplasm of the cells in NP and inner AF became shrunken, and staining of the ECM began subtly to lighten (Figure 1(d)). Alcian blue staining intensity can reflect the content of glycosaminoglycans in cartilage or cartilage-like tissue [24–26]. As is shown in Figure 1(e), both the NP and inner AF exerted 0.5 MPa hydrostatic pressure had the highest intensity of alcian blue staining. When the pressure level increased over 0.8 MPa, the staining intensity was declined gradually with the rise of pressure loading (Figure 1(e)). Additionally, quantificational sGAG assessment further proved that both the NP and inner AF under 0.5 MPa hydrostatic pressure had the highest content of sGAG (Figures 1(f) and 1(g)). When pressure level reached ≥0.8 MPa, the content of sGAG decreased gradually in the NP tissues (Figure 1(f)). However, 0.8 MPa hydrostatic pressure did not decline the content of sGAG compared with the group of tissue exerted 0.5 MPa hydrostatic pressure in inner AF (Figure 1(g)). The results above indicated that low-loading physiological hydrostatic pressure was beneficial for glycosaminoglycans synthesis of notochordal NP and fibrocartilaginous inner AF. However, high-magnitude hydrostatic pressure markedly attenuated the matrix sGAG synthesis in both notochordal NP and fibrocartilaginous inner AF. Noticeably, the diversity of cell morphology was more evident in NP tissues, whereas the diversity of glycosaminoglycans content was more significant in inner AF tissues under different levels of hydrostatic pressure.

### 3.2. Effect of Hydrostatic Pressure on Cellular Survival of the Rabbit Notochordal NP and Fibrocartilaginous Inner AF

**Figure 2:**
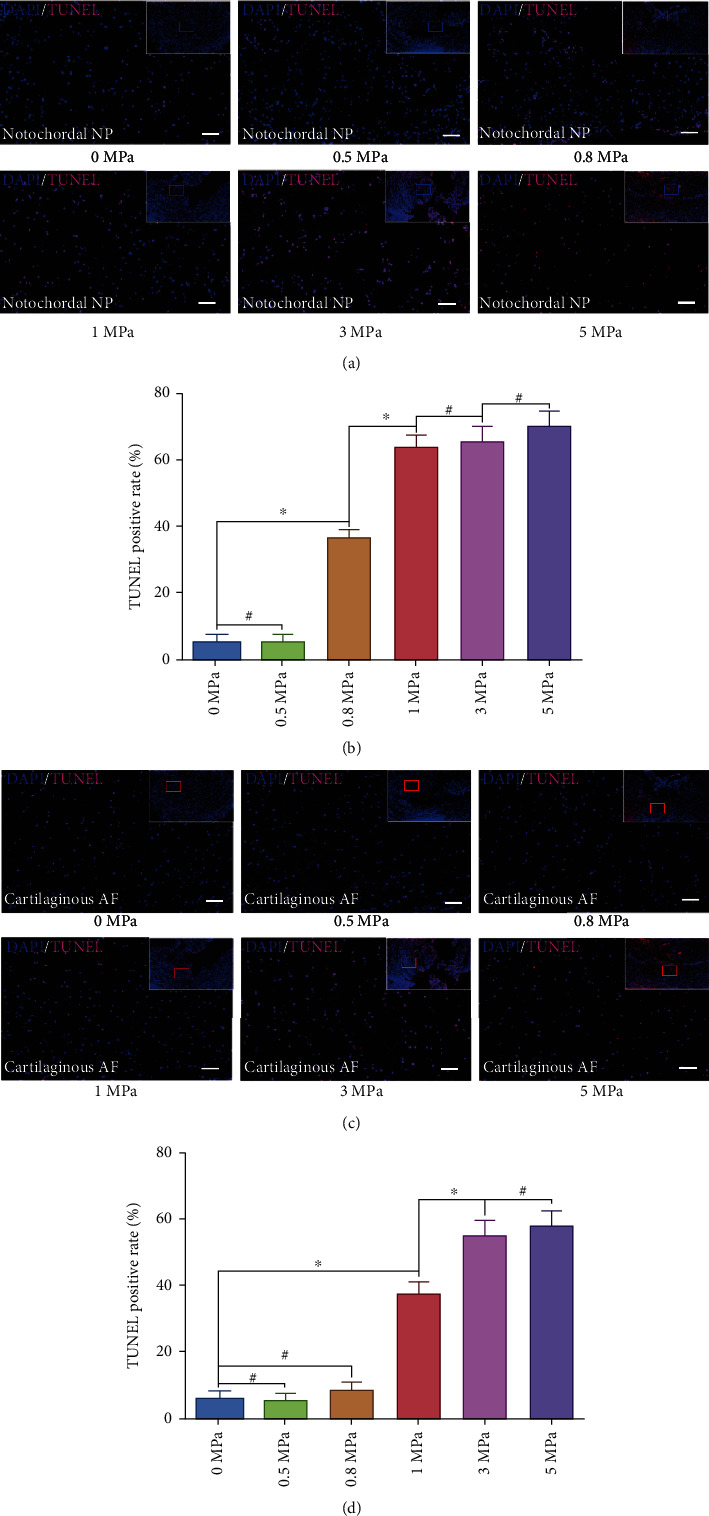
Effect of hydrostatic pressure on cellular survival of the rabbit notochordal NP and fibrocartilaginous inner AF: (a) fluorescent TUNEL staining of the notochordal NP under graded hydrostatic pressure (200x); (b) statistic analysis of TUNEL positive rate of the notochordal NP under graded hydrostatic pressure; (c) fluorescent TUNEL staining of the fibrocartilaginous inner AF under graded hydrostatic pressure (200x); (d) statistic analysis of TUNEL positive rate of the fibrocartilaginous inner AF under graded hydrostatic pressure. ^∗^*P* < 0.05. ^**#**^*P* > 0.05. Scale bar = 100 *μ*m.

Based on the former overall morphology analysis, we further detected the cell survival rate in notochordal NP and fibrocartilaginous inner AF tissues via fluorescent TUNEL staining. The experimental results revealed that ≥0.8 MPa hydrostatic pressure significantly increased the cell apoptosis rate in NP tissues (Figures 2(a) and 2(b)), while the apoptosis rate of inner AF cells was markedly aggravated when exerted ≥0.5 MPa hydrostatic pressure (Figures 2(c) and 2(d)). However, there was no significant difference in TUNEL positive rate between the 3.0 MPa and 5.0 MPa groups both for the NP and inner AF (Figures 2(b) and 2(d)), which indicated that 3.0 MPa was the ultimate hydrostatic pressure level for the NP and inner AF cell survival.

### 3.3. Effect of Hydrostatic Pressure on the Synthesis of Extracellular Aggrecan of the Rabbit Notochordal NP and Fibrocartilaginous Inner AF

**Figure 3:**
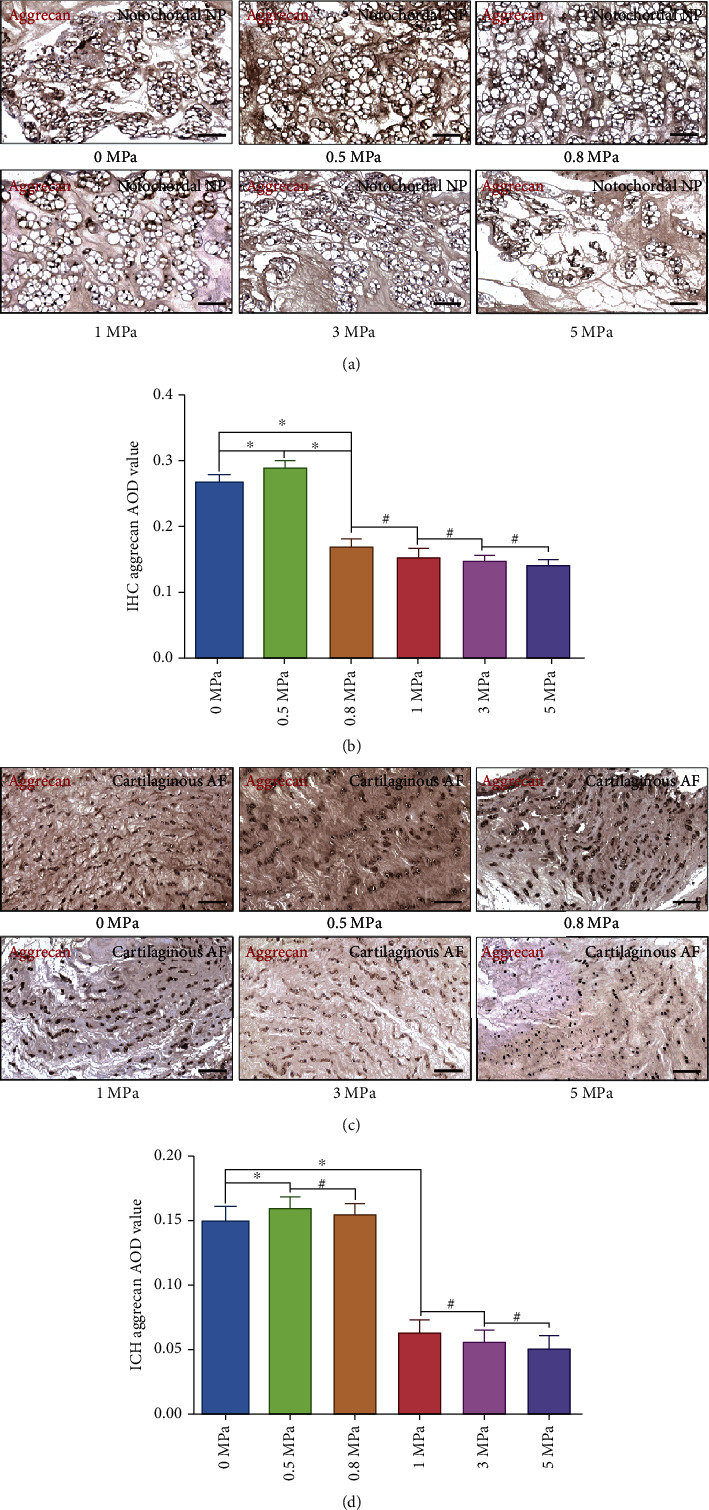
Effect of hydrostatic pressure on synthesis of aggrecan of the rabbit notochordal NP and fibrocartilaginous inner AF: (a) aggrecan IHC staining of the notochordal NP under graded hydrostatic pressure (200x); (b) statistic analysis of IHC aggrecan AOD value of the notochordal NP under graded hydrostatic pressure; (c) aggrecan IHC staining of the fibrocartilaginous inner AF under graded hydrostatic pressure (200x); (d) statistic analysis of IHC aggrecan AOD value of the fibrocartilaginous inner AF under graded hydrostatic pressure. ^∗^*P* < 0.05. ^**#**^*P* > 0.05. Scale bar = 100 *μ*m.

The proteoglycan complex, predominantly aggrecan, is responsible for the hydrophilic nature of the NP, which can exert a mechanical influence upon NP cells through regulating nutrient transport, hydration, and swelling pressure [1, 27]. Additionally, inner AF represents a transitional zone and contains great amounts of aggrecan as it is subjected to compressive loads transferred from NP [1, 28]. Therefore, content of aggrecan is an important indicator to evaluate the degeneration degree of IVD. As is shown in Figure 3, both NP and inner AF under 0.5 MPa hydrostatic pressure had the highest content of matrix aggrecan. When the pressure level reached ≥0.8 MPa, the content of aggrecan declined gradually with the rise of pressure loading in the NP tissues (Figures 3(a) and 3(b)). However, 0.8 MPa hydrostatic pressure did not decline the synthesis of aggrecan in inner AF tissues compared with the group of tissues exerted ≤0.5 MPa hydrostatic pressure (Figures 3(c) and 3(d)). In contrast, the expression of aggrecan in inner AF was significantly attenuated when hydrostatic pressure exceeded 1.0 MPa (Figures 3(c) and 3(d)).

### 3.4. Effect of Hydrostatic Pressure on the Synthesis of Type I and Type II Collagen of the Rabbit Notochordal NP and Fibrocartilaginous Inner AF

**Figure 4:**
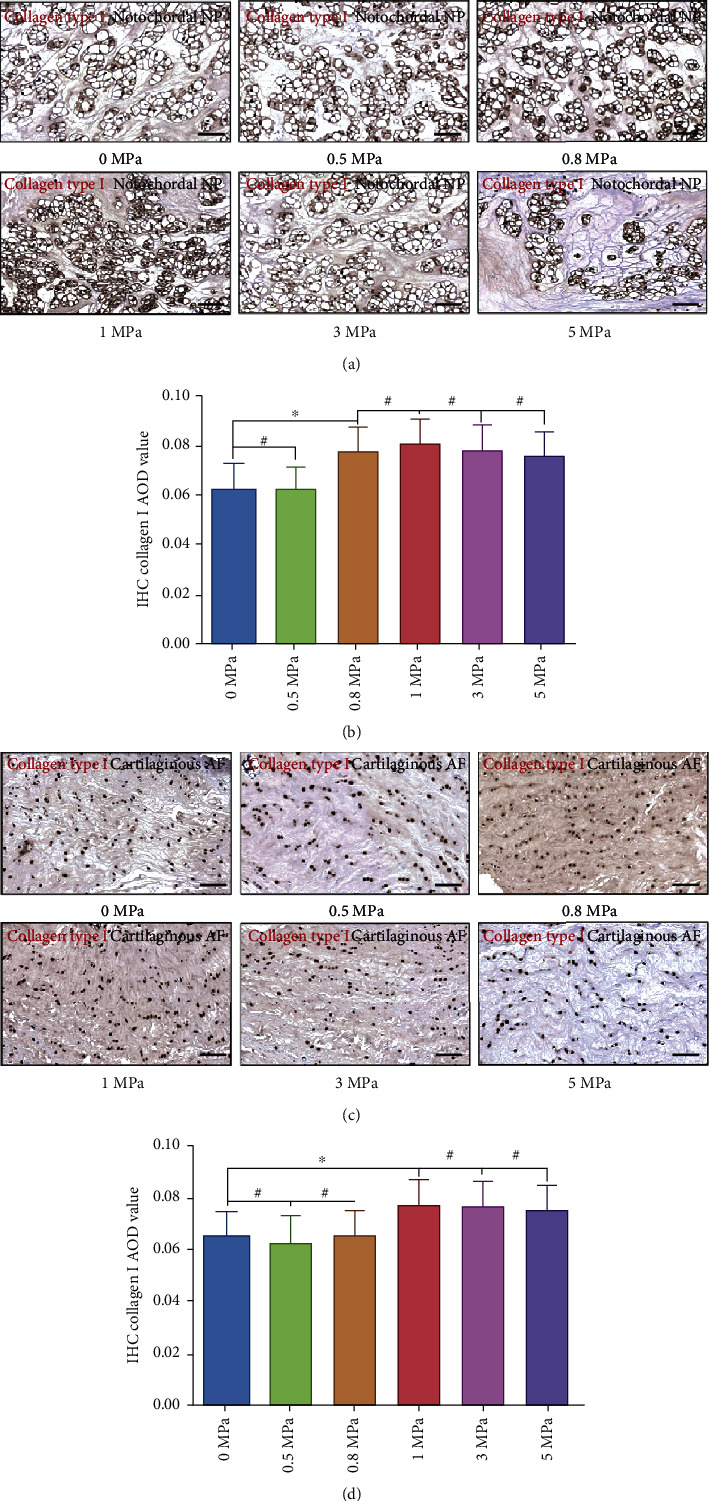
Effect of hydrostatic pressure on synthesis of collagen type I of the rabbit notochordal NP and fibrocartilaginous inner AF: (a) collagen type I IHC staining of the notochordal NP under graded hydrostatic pressure (200x); (b) statistic analysis of IHC collagen type I AOD value of the notochordal NP under graded hydrostatic pressure; (c) collagen type I IHC staining of the fibrocartilaginous inner AF under graded hydrostatic pressure (200x); (d) statistic analysis of IHC collagen type I AOD value of the fibrocartilaginous inner AF under graded hydrostatic pressure. ^∗^*P* < 0.05. ^**#**^*P* > 0.05. Scale bar = 100 *μ*m.

**Figure 5:**
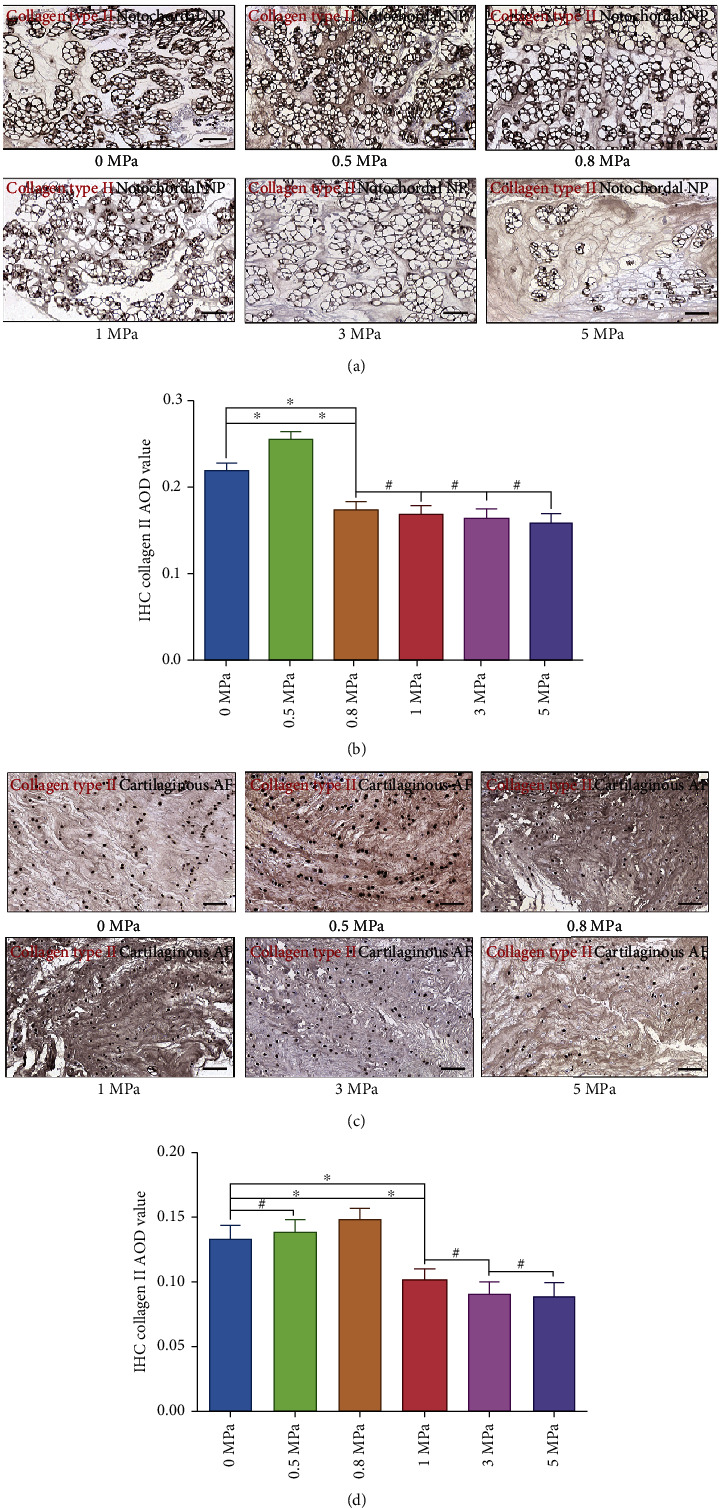
Effect of hydrostatic pressure on synthesis of collagen type II of the rabbit notochordal NP and fibrocartilaginous inner AF: (a) collagen type II IHC staining of the notochordal NP under graded hydrostatic pressure (200x); (b) statistic analysis of IHC collagen type II AOD value of the notochordal NP under graded hydrostatic pressure; (c) collagen type II IHC staining of the fibrocartilaginous inner AF under graded hydrostatic pressure (200x); (d) statistic analysis of IHC collagen type II AOD value of the fibrocartilaginous inner AF under graded hydrostatic pressure. ^∗^*P* < 0.05. ^**#**^*P* > 0.05. Scale bar = 100 *μ*m.

Besides the abundant content of proteoglycans, the NP and inner AF also contain randomly organized collagen, mainly type II, which forms a fibril mesh-like framework to support structures [28]. However, with the development of degeneration, the IVD exhibits more characteristics of fibrous tissues and resembles ligament and tendon with large amounts of type I collagen fibrils [23, 28]. Thus, we further detected the expression level of collagen types I and II via IHC analysis. As is shown in Figure 4, the expression of matrix collagen type I was enhanced when the hydrostatic pressure reached ≥0.8 MPa in NP tissues (Figures 4(a) and 4(b)). In inner AF, the expression level of collagen type I was enhanced by ≥1.0 MPa hydrostatic pressure (Figures 4(c) and 4(d)). In contrast, the expression of collagen type II in the NP was enhanced by 0.5 MPa hydrostatic pressure but attenuated by ≥0.8 MPa hydrostatic pressure (Figures 5(a) and 5(b)), whereas the expression of collagen type II in the inner AF was enhanced by hydrostatic pressure ranged 0.5-0.8 MPa but attenuated by hydrostatic pressure over 1.0 MPa (Figures 5(c) and 5(d)).

### 3.5. Differential Expression of the N-CDH and Integrin in the Rabbit Notochordal NP and Fibrocartilaginous Inner AF

**Figure 6:**
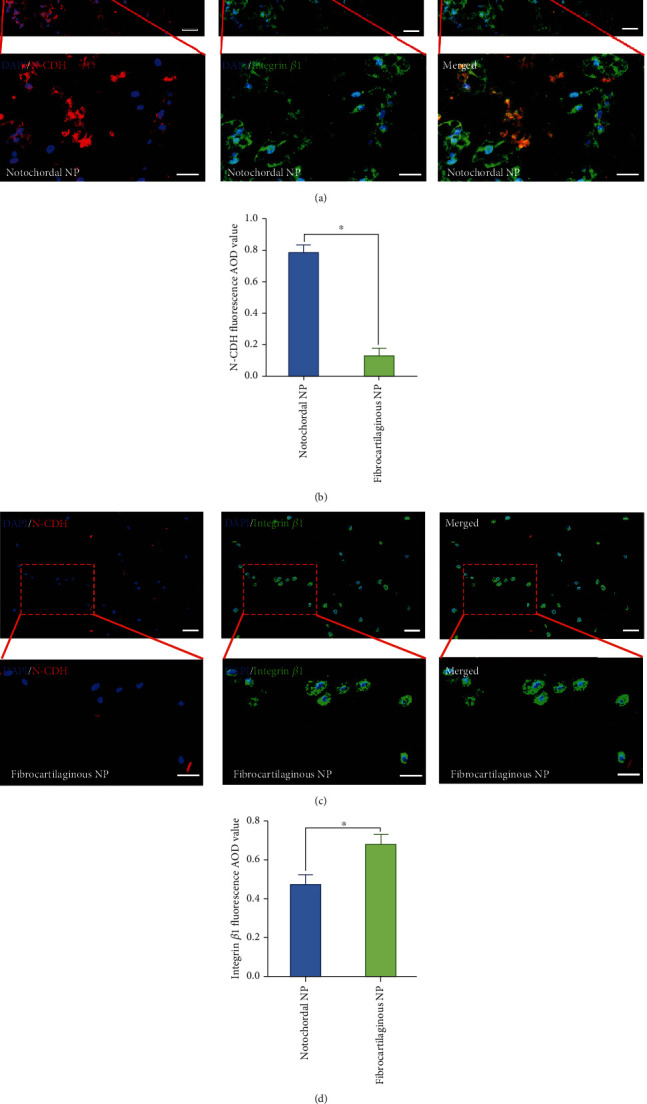
Differential expression of N-CDH and integrin *β*1 in the rabbit notochordal NP and fibrocartilaginous inner AF: (a) N-CDH (red) and integrin *β*1 (green) fluorescent staining of rabbit notochordal NP (200x); (b) statistic analysis of fluorescent N-CDH AOD value of the notochordal NP and fibrocartilaginous inner AF; (c) N-CDH (red) and integrin *β*1 (green) fluorescent staining of rabbit fibrocartilaginous inner AF (200x); (d) statistic analysis of fluorescent integrin *β*1 AOD value of the notochordal NP and fibrocartilaginous inner AF. ^∗^*P* < 0.05. Scale bar = 100 *μ*m.

To further figure out the possible molecular mechanism causing the differential response to the hydrostatic pressure in notochordal NP and fibrocartilaginous inner AF, we detected the expression of N-CDH and integrin *β*1, which are acknowledged mechanosensitive factors in IVD cells [29]. Double fluorescent label of N-CDH (red fluorescence) and integrin *β*1 (green fluorescence) indicated that the expression of N-CDH was much higher in the notochordal NP than the inner AF (Figures 6(a)–6(c)). However, the expression of integrin *β*1 was relatively higher in the inner AF than the notochordal NP (Figures 6(a), 6(c), and 6(d)).

### 3.6. Physiological Hydrostatic Pressure Affects the IVD Cell Survival and ECM Homeostasis via YAP/TAZ Pathway

**Figure 7:**
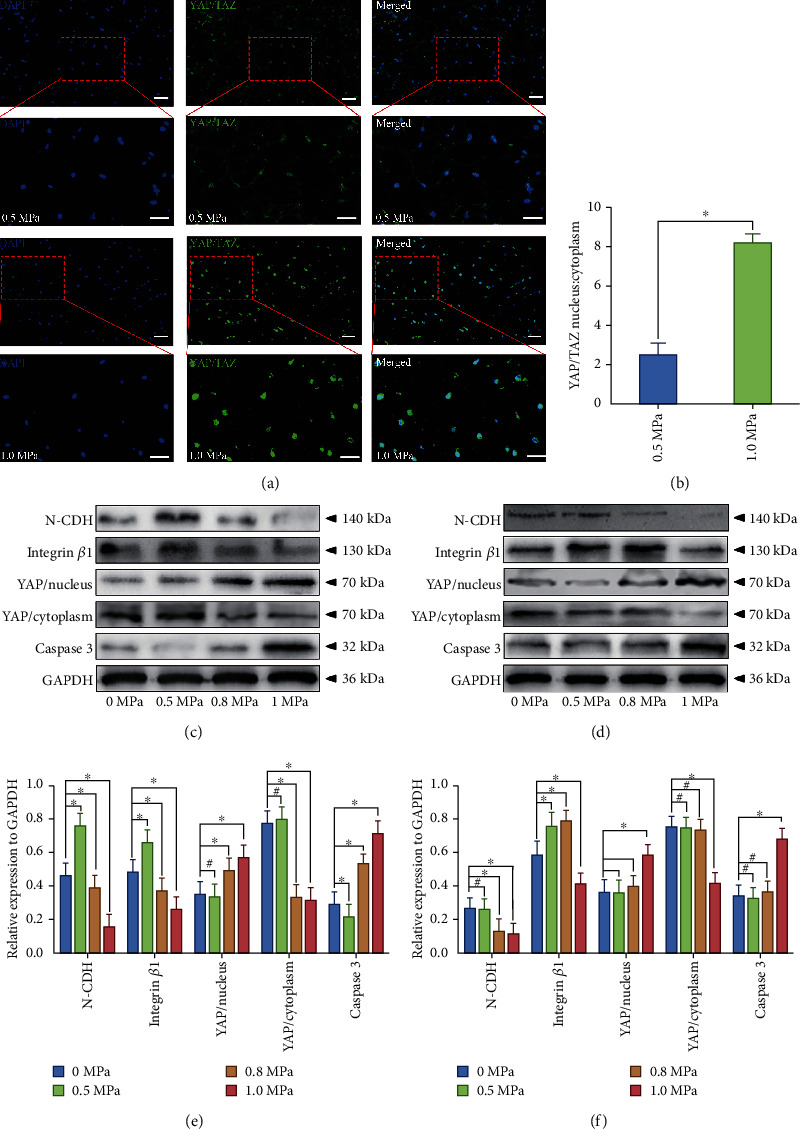
Effect of hydrostatic pressure on YAP/TAZ pathway-mediated apoptosis of the rabbit notochordal NP and fibrocartilaginous inner AF: (a) YAP/TAZ complex (green) fluorescent staining of the fibrocartilaginous inner AF under low-loading (0.5 MPa) or high-magnitude (1.0 MPa) physiological hydrostatic pressure (200x); (b) statistic analysis of YAP/TAZ complex nucleus/cytoplasm distribution; (c) western blotting analysis of the expression levels of N-CDH, integrin *β*1, and YAP/TAZ-mediated apoptosis markers (YAP/nucleus, YAP/cytoplasm, Caspase3) of the notochordal NP under graded hydrostatic pressure; (d) western blotting analysis of the expression levels of N-CDH, integrin *β*1, and YAP/TAZ-mediated apoptosis markers (YAP/nucleus, YAP/cytoplasm, Caspase3) of the fibrocartilaginous inner AF under graded hydrostatic pressure; (e) statistic analysis of the western blots of the notochordal NP under graded hydrostatic pressure; (f) statistic analysis of the western blots of the fibrocartilaginous inner AF under graded hydrostatic pressure. ^∗^*P* < 0.05. ^**#**^*P* > 0.05. Scale bar = 100 *μ*m.

YAP and its paralogue PDZ-binding motif (TAZ) known as the regulator of IVD cell survival were related to N-CDH and integrin-mediated cell-cell and cell-matrix attachment [30]. As is shown in Figures 7(a) and 7(b), the nucleus, cytoplasm proportion of YAP/TAZ was significantly elevated in the 1.0 MPa group compared with the 0.5 MPa group. In addition, the western blots in Figures 7(c) and 7(e) revealed the differential protein expression in the notochordal NP under graded hydrostatic pressure, while the western blots in Figures 7(d) and 7(f) revealed the differential protein expression in the fibrocartilaginous inner AF under graded hydrostatic pressure. As is shown in Figures 7(c) and 7(e), the expression of N-CDH and integrin *β*1 in NP was elevated under 0.5 MPa hydrostatic pressure but declined under ≥0.8 MPa hydrostatic pressure. The expression of YAP in the nucleus and Caspase3 was also enhanced by ≥0.8 MPa hydrostatic pressure in NP (Figures 7(c) and 7(e)). Correspondingly, the expression of YAP in the cytoplasm was attenuated by ≥0.8 MPa hydrostatic pressure in NP (Figures 7(c) and 7(e)). The blots in Figures 7(d) and 7(f) showed that the expression of N-CDH in AF declined under ≥0.8 MPa hydrostatic pressure. The expression of integrin *β*1 was enhanced in AF under the hydrostatic pressure ranged 0.5-0.8 MPa but declined under ≥1 MPa hydrostatic pressure (Figures 7(d) and 7(f)). The expression of YAP in nucleus and Caspase3 was enhanced by ≥1.0 MPa hydrostatic pressure in AF (Figures 7(c) and 7(e)). Correspondingly, the expression of YAP in cytoplasm was attenuated by ≥1.0 MPa hydrostatic pressure in AF (Figures 7(c) and 7(e)).

## 4. Discussion

It is commonly held that the overloaded compressive force applied to the IVD is one of the causes of IVD degeneration [31, 32], whereas the proper physiological pressure is beneficial for maintaining the cell viability and ECM homeostasis of IVD [1, 2, 10]. There are still some contradictions in the prior studies about the effects of different pressure loading methods or intensities on the biological behavior of the IVD. The majority holds that the cells in NP and inner AF tissues mainly suffered hydrostatic pressure, because when a compressive force is applied to the IVD, the hydrostatic pressure within NP and inner AF is increased [9, 27]. Hence, in the present study, we used the self-developed hydrostatic pressure bioreactor based on a pressure-transmitting mode achieved by a slight deformation of a flexible membrane in a completely sealed stainless steel chamber, to imitate in vivo microenvironment for NP and inner AF tissues. And the tissues' responses to the graded hydrostatic pressure were observed via histological and cytologic analysis. The results of our study indicated that low-loading physiological hydrostatic pressure (0.5 MPa) was beneficial for cellular survival and ECM homeostasis both in the notochordal NP and fibrocartilaginous inner AF tissues, whereas high-magnitude dynamic hydrostatic pressure (≥1.0 MPa) aggravated cell apoptosis and breakdown of ECM both in the notochordal NP and fibrocartilaginous inner AF. However, there was still some diversity in the sensitivity to the hydrostatic pressure between notochordal NP and fibrocartilaginous inner AF. Specifically, the cell apoptosis rate was markedly increased in the NP tissue suffered ≥0.8 MPa hydrostatic pressure. In contrast, the cell apoptosis in the inner AF tissue was significantly increased by ≥1.0 MPa hydrostatic pressure. Additionally, the expression of sGAG, aggrecan, and type II collagen was consistent with the trend of the cell apoptosis rate in the notochordal NP and fibrocartilaginous inner AF. Furthermore, we detected the expression of N-CDH and integrin *β*1, two acknowledged mechanosensitive factors in regulating the phenotype and function of NP as well as AF cell. We found that N-CDH was expressed more prominently in NP, while integrin *β*1 was expressed more prominently in AF. Our results also revealed that YAP/TAZ localization predominantly transferred to nuclear and triggered cell apoptosis via Hippo-YAP/TAZ pathway when the cells suffered high-magnitude hydrostatic pressure (0.8 MPa for NP; 1.0 MPa for AF). These results indicated that notochordal NP cells were more sensitive to the changes in pressure comparing with the fibrocartilaginous inner AF cells. The potential molecular mechanism was related to the differential expression of mechanosensitive factors and Hippo-YAP/TAZ-associated cell death.

N-CDH was a notochord-associated gene and also regarded as an NP-specific biomarker [33]. Embryonically, the NP originated from the notochord, while the AF arises from the mesenchyme [1, 34]. Thus, the expression of N-CDH was more prominent in notochordal NP. However, notochordal cells are replaced by chondrocyte-like cells of unknown provenance before skeletal maturity in humans [1, 2, 5, 34]. Some studies declared that the lack of hydrostatic pressure or overloaded pressure could induce the disappearance of notochordal cells in the NP tissue [15–17]. The physiological pressure magnitudes in IVD tissue have been estimated to be approximately 0.1 to 0.5 MPa under low-loading conditions and with values as high as 1.0 to 3.0 MPa under extreme loading conditions [35]. Our research further confirmed that the medium-loading degree of hydrostatic pressure (0.8-1.0 MPa) could even induce the degeneration of notochordal NP via attenuating the expression of N-CDH, exacerbating cell death and catabolism of ECM, especially aggrecan and type II collagen fibril. Notochordal cell was reported to have a high potential for proteoglycan production and suppressing cell apoptosis via secreting some functional factors [14, 34, 36]. This is why the time that notochordal NP began to disappear coincided with the occurrence of morphologic signs of disc degeneration [34, 36]. Thus, with the accumulation of compressive loading on the human spine, the degeneration of notochordal NP was unstoppable.

Nevertheless, we also observed that the inner AF exhibited relatively higher expression of integrin *β*1 and greater tolerance to the physiological medium loading degree of hydrostatic pressure. Detailedly, the morphology of the chondrocyte-like cells and the ECM homeostasis of the inner AF under medium loading degree of hydrostatic pressure (0.8-1.0 MPa) revealed no markedly difference comparing with those cultured under atmospheric pressure. However, when the pressure level increased to the high-magnitude degree (>1.0 MPa), the cell death rate was enhanced, and the breakdown of ECM homeostasis was triggered. The characteristic phenotype of the cell in mature human NP displayed a rounded, chondrocyte-like morphology, and secreted ECM macromolecules, which more resembled the typical phenotype of the inner AF cells in nonerect walking animals, such as rabbit or dog [37]. As the human spine suffered more magnitude and frequency of axial compressive loading than the nonerect walking animals, the cell type in human IVD should be more tolerant to compression stress [6]. A previous study indicated that N-CDH and integrin-mediated adhesive interactions modulated YAP-TAZ associated mechanosensing and fate commitment of cells [19]. Noticeably, N-CDH mainly mediated softer cell-cell mechanosensing signal, while integrin-mediated stiffer cell-ECM mechanosensing signal [19]. Our research authenticated that the cell viability and ECM producing ability of the cartilaginous inner AF were superior to the notochordal NP under medium or high loading degree of hydrostatic pressure. The possible molecular mechanism was related to the high expression of integrin and attenuated YAP/TAZ-associated cell death. Although the transition from the notochordal NP to the cartilaginous one was considered as a morphologic sign of IVD degeneration, maintenance of the normal cartilaginous phenotype and matrix producing function was more meaningful for exploring human IVD regeneration strategies.

Although there are some novel findings in the present study, several limitations also exist. Firstly, the tissue samples in the present study were cultured under normoxic conditions, because of the lack of hypoxia-culture settings in our bioreactor. This condition is different from the physiological hypoxic condition in which the NP and inner AF cells live [1]. Our team has been working on updating the devices of our bioreactor and tries to imitate a more bionic microenvironment for the in vitro IVD tissue culture. Secondly, the pressure setting precision was relatively rough for the IVD tissue, because this generation of hydrostatic pressure bioreactor was designed for imitating the in vivo condition for all musculoskeletal junctions, such as meniscus and articular cartilage. Thus, the loading device was designed to exert high-magnitude pressure (maximal value = 10 MPa) for the chamber, which limited the accuracy regulating the function of the bioreactor. We have been working on developing a low-pressure loading device with more accurate pressure setting system for NP culture and tries to figure out a more precise data about the effect of hydrostatic pressure on NP biological behavior in future studies.

**Figure 8:**
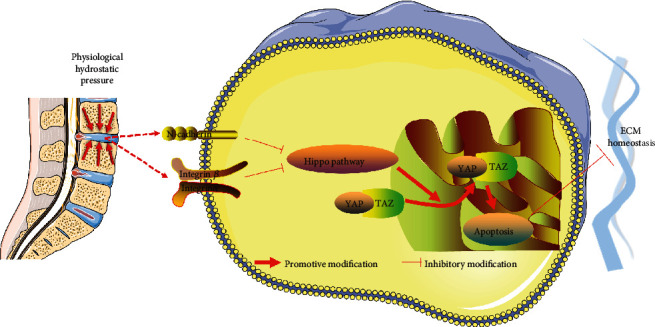
Schematic diagram shows the potential mechanism of physiological hydrostatic pressure effects the IVD cell survival and ECM homeostasis: low-loading physiological hydrostatic pressure activates mechanosensitive factors N-CDH and integrin *β*1, which inhibit Hippo-YAP/TAZ pathway-mediated cell apoptosis and ECM catabolism.

To conclude, our study reveals that low-loading physiological hydrostatic pressure is beneficial for cell survival and ECM homeostasis in notochordal NP and fibrocartilaginous inner AF. Its possible mechanism is related to the upregulated protective mechanosensing factors (N-CDH and integrin *β*1) under low-loading hydrostatic pressure (Figure 8), whereas high-magnitude hydrostatic pressure aggravates the breakdown of ECM homeostasis in NP and inner AF via downregulating N-CDH and integrin *β*1, which attenuated the inhibitory action of YAP/TAZ-mediated cell apoptosis and ECM catabolism. Moreover, inner AF exhibits a more prominent expression of integrin *β*1 and tolerance to the medium-loading degree of physiological hydrostatic pressure than the notochordal NP. This study may provide a better understanding of the regulatory role of hydrostatic pressure on the cell survival and matrix metabolism of the IVD and more substantial evidence for using hydrostatic pressure bioreactor in exploring the disc degeneration mechanism as well as regeneration strategies.

## Data Availability

The data used to support the findings of this study are available from the corresponding author upon request.
